# Gestational diabetes mellitus: a qualitative study of lived experiences of South Asian immigrant women and perspectives of their health care providers in Melbourne, Australia

**DOI:** 10.1186/s12884-021-03981-5

**Published:** 2021-07-09

**Authors:** Mridula Bandyopadhyay

**Affiliations:** Department of Obstetrics & Gynaecology, Faculty of Medicine, Dentistry and Health Sciences, The University of Melbourne, Parkville Victoria, 3052 Australia

**Keywords:** Gestational diabetes mellitus, Self-management, South Asia, Immigrant women, Health care providers, Australia

## Abstract

**Background:**

South Asian women are at a high risk of developing gestational diabetes mellitus than other women in Australia. Gestational diabetes affects up to 14–19% of all pregnancies among South Asian, South East Asian, and Arabic populations placing women at risk of adverse pregnancy outcomes. Although, gestational diabetes resolves after childbirth, women with gestational diabetes are up to seven times more likely to develop type 2 diabetes within five to ten years of the index pregnancy. Increasingly, South Asian women are being diagnosed with gestational diabetes in Australia. Therefore, we aimed to gain a better understanding of the lived experiences of South Asian women and their experiences of self-management and their health care providers’ perspectives of treatment strategies.

**Methods:**

Using an ethnographic qualitative research methodology, semi-structured one-on-one, face-to-face interviews were conducted with 21 health care providers involved in gestational diabetes management and treatment from the three largest tertiary level maternity hospitals in Melbourne, Victoria, Australia. In-depth interviews were conducted with 23 South Asian women post diagnosis between 24–28 weeks gestation in pregnancy.

**Results:**

Health care providers had challenges in providing care to South Asian women. The main challenge was to get women to self-manage their blood glucose levels with lifestyle modification. Whilst, women felt self-management information provided were inadequate and inappropriate to their needs. Women felt ‘losing control over their pregnancy’, because of being preoccupied with diet and exercise to control their blood glucose level.

**Conclusions:**

The gestational diabetes clinical practice at the study hospitals were unable to meet consumer expectations. Health care providers need to be familiar of diverse patient cultures, rather than applying the current ‘one size fits all’ approach that failed to engage and meet the needs of immigrant and ethnic women. Future enabling strategies should aim to co-design and develop low Glycaemic Index diet plans of staple South Asian foods and lifestyle modification messages.

**Supplementary Information:**

The online version contains supplementary material available at 10.1186/s12884-021-03981-5.

## Background

Women from South Asia (Indian sub-continent) have a high risk of developing gestational diabetes mellitus (GDM) [1–3], and are almost four times more likely than other women in Australia to develop GDM. It is defined as glucose intolerance with onset or first recognition during pregnancy [4] and affects up to 14–19% of all pregnancies in some populations, such as South Asians, South East Asians, and Arabic, placing women at risk of adverse pregnancy outcomes [5, 6]. The consequences of GDM on maternal and fetal health are significant [4, 7]. Though GDM resolves after childbirth, women with GDM are up to seven times more likely to develop type 2 diabetes within 5–10 years after the index pregnancy compared to women with normoglycaemic pregnancy [8, 9]. Despite this, there is limited literature on South Asian immigrant experiences in Australia. This prompted our interest in exploring South Asian women’s experiences of living with GDM and self-management—the first study in Australia that highlighted the significant difficulty South Asian women experienced in self-management of GDM. Therefore, we felt it was important to investigate health care providers perspectives of their consultations with South Asian women on GDM management as no previous studies reported on this.

Informed by our previous study [8] we felt it was important to gain a better understanding of both health care providers’ perspectives and women’s own experiences of diagnosis and self-management of GDM. As self-management of GDM places emphasis on diet and exercise primarily, we recognised that our respondents’ opinions and experiences could possibly be used to inform broader understandings of the facilitators and challenges observed in GDM management. Thus, this study aimed to complement the emerging body of qualitative studies on GDM in Australia. Currently, there is an upsurge of interest in the field of GDM among public health professionals and health care providers alike, but knowledge on GDM is still in its infancy and developing. Hence, our study was designed to contribute and build new knowledge base on GDM literature.

In Australia, the recommended diet for women with GDM is on blood glucose control, that is, having lower glycemic index diet and distributing carbohydrate intake throughout the day [10]. The advice places emphasis on changing the food composition patterns and the quantity of food intake in pregnancy. For any dietary advice to be effective, individualized dietary plans that considers respondents personal and cultural preferences, beliefs and practices, is important [11, 12]. Our objective was to inform the delivery of dietary advice particularly for women with GDM, but also for the health care providers who deliver these health education messages at diagnosis and follow-up.

## Method

This study was undertaken in Melbourne which contains the largest concentration of South Asian communities in Australia. Most South Asians in Melbourne originated from India, Sri Lanka, Bangladesh, Pakistan, and Fiji. Although there is some diversity among them in terms of religion and language, they share similarities in terms of food and diet and cultural practices followed in the antenatal and postnatal period [13]. An ethnographic qualitative methodology was used to elicit information and collect data, including observation of participants in both group settings and at an individual level [14]. This approach was used to address the social determinants of health within participants social contexts.

### Recruitment and sample

Following ethics approval from the La Trobe University Human Research Ethics Committee and the respective hospital Research Ethics Committees, participants were recruited from three maternity hospitals in Melbourne where routine screening and diagnosis of GDM usually occurs between 24–28 weeks of gestation with an oral glucose tolerance test (Table 1). A high proportion of South Asian women present for maternity care at these maternity hospitals. Respective hospital-based diabetes nurse educator (DNE)—White English-speaking Australian—approached ethnic South Asian women (identified by country of birth and self-identified) aged 18 years and above diagnosed with GDM at the diabetes clinic and invited them to participate in the study between June and December 2011. Similarly, from the same maternity hospitals’ health care providers, including obstetrician and gynaecologists, endocrinologists, physicians, midwives, diabetes nurse educators, dietitians, physiotherapists, and obstetric/medical registrars involved in routine GDM treatment and management were invited to participate in the study by the same DNE between July and December 2011.

**Table 1:** Gestational diabetes mellitus diagnostic and management criteria at the study hospitals

Criteria	Hospital A	Hospital B	Hospital C
Diagnostic	^a^Fasting ≥ 5.5—6.1 mmol/L ^b^2 h ≥ 8 mmol/L	^a^Fasting ≥ 5.1—6.9 mmol/L ^b^2 h ≥ 8.5 mmol/L	^a^Fasting ≥ 5.1 – 6.5 mmol/L 1 h ≥ 10.0 mmol/L ^b^2 h ≥ 8.5 mmol/L
Management	^a^Fasting ≤ 5.0 mmol/L Post postprandial ≤ 6.7	^a^Fasting ≤ 5.4 Post postprandial ≤ 7.0	^a^Fasting ≤ 5.5 Post postprandial ≤ 6.5

^a^Fasting: oral glucose tolerance test (OGTT)

^b^2 h: glucose tolerance test (GTT)

At recruitment participants were provided with a plain language information and consent form in English. A translated version (in different South Asian languages) was provided if requested, explaining the research project, its procedure and risks to help decide if they wanted to take part in the study. The principal researcher (not a health care provider nor an employee at any of the maternity hospitals) was available at recruitment to answer any questions relating to the study and consent. Recruitment of both health care providers and GDM diagnosed women continued until no new themes emerged from data analysis [15].

Semi-structured interviews with open-ended questions were conducted with 21 health care providers (Additional file 2) involved in GDM treatment and management from the three largest tertiary level hospitals in Melbourne, Victoria, Australia (Table 2). Interviews started with an open-ended question inviting health care providers to describe their experiences of consultations with South Asian women. To achieve a representative sample of South Asian women, Indian, Pakistani, Sri Lankan, Bangladeshi, Indian-Fijian, Indian-Australian women diagnosed with GDM were purposively sampled. Twenty-three GDM diagnosed women consented to take part in the study (Table 3).

**Table 2:** Characteristics of health care professionals

Health care professionals	
Diabetes Nurse Educators	8
Dietitians	4
Obstetric and endocrinology fellow	3
Obstetrician and gynaecologist	3
Endocrinologist	3
**Total**	**21**

**Table 3:** Socio-demographic and other characteristics of GDM diagnosed women

Country of birth	India	13
	Pakistan	5
	Sri Lanka	3
	Fiji	1
	Australia (2^nd^ generation Indian)	1
Length of residence in Australia	< 1 year to 18 years	Median 4.5 years
Age range	24 – 38 years	Median 29 years
Marital status	Married	23
Education	M.B.B.S	1
	Nursing	2
	Dentistry	1
	Postgraduate	10
	Graduate	7
	Year 12	1
	Vocational (TAFE)	1
Employment status	Full time	11
	Homemaker	12
Occupation	Public service	2
	IT professional	2
	Doctor	1
	Nurse	1
	Dentist	1
	Teacher	1
	Hairdresser	1
	Small business owner	2
Religion	Hinduism	7
	Islam	6
	Christianity	4
	Buddhism	3
	Sikhism	3
Parity	1^st^ Pregnancy	9
	2^nd^ Pregnancy	12
	3^rd^ Pregnancy	1
	7^th^ Pregnancy	1
Previous pregnancy GDM status	Yes	7
Insulin status in current pregnancy	Yes (within a couple of weeks’ post diagnosis)	16
	No (managing with diet and exercise at the time of interview)	7
Early diagnosis of GDM	Yes (diagnosed at: 9, 12, 14, & 22 weeks’ gestation)	4
Family history of diabetes	Yes	19
	No	4
Awareness of type 2 diabetes mellitus	Yes	17
	No	6
Awareness of GDM before current pregnancy	Yes	10
	No	13
Weight issues pre-pregnancy	Yes	18
	No	5
Weight gain in current pregnancy	Yes	13
	No	10
Pregnancy weight issues	Yes	18
	No	5
Diet	Vegetarian	8
	Non-vegetarian	15

### Data collection

Written consent was obtained from each participant before any data collection commenced. One-on-one face-to-face interviews were conducted in 2011 with 23 GDM diagnosed South Asian women. Respondents were interviewed in their preferred language (English or Hindi/Urdu) by the principal multilingual researcher (MB). Interviews started with an open-ended question inviting women to narrate their experiences of GDM. Interviews were informed by interview guidelines (Additional file 1), which were revised in view of emerging themes. Relevant themes covered during the interviews included: food and eating habits during a typical day and during pregnancy in particular, including the kinds and quantity of foods eaten and when (time); any changes in diet since migration and diagnosis of GDM; perceived barriers and facilitators to dietary changes; role in food purchase and preparation; perceptions of consuming special foods in pregnancy; availability of ‘special pregnancy foods’ in Australia; social and symbolic role of food in pregnancy and its relationship with health and well-being; and perceived impact of diet and dietary modifications after GDM diagnosis on self -health, health of baby and identity of being a South Asian. We explored women’s perception of barriers and facilitators to diet modification, and the social and cultural factors informing their narratives.

One-on-one face-to-face interviews with 21 health care providers covered themes around: health care providers’ perceptions of treating and caring for GDM diagnosed South Asian women, perceptions of difficulties/problems faced in providing care, type of advice and health messages provided for self-management, frequency of contact, and style and type of communication with diagnosed women. Data analysis was carried out alongside data collection to facilitate identification of emerging themes, which were explored in all subsequent interviews.

Interviews took place at a place and time convenient to the participants and averaged around 40–45 min with the care providers’ and an hour with the diagnosed women. Interviews were conducted at the diabetes clinic consulting rooms and were digitally recorded with consent. All interviews with health care providers were conducted in English. Twenty interviews with diagnosed women were conducted in English and three in Hindi/Urdu. The interviews conducted in Hindi/Urdu were translated verbatim by MB into English. Interviews conducted in English were transcribed verbatim by a professional transcription company. To ensure rigour and to highlight potential areas of misunderstanding MB was assisted by a multilingual professional translator to unpack and understand the meaning of certain concepts, which was cross-checked with available literature.

### Data analysis

Transcripts were read repeatedly and cross-compared by team members using the constant comparative method [15] and discussed to identify analytical categories and key themes. At this stage, field notes including observation data was combined in the analysis. Categories were then organised based on emerging themes and subthemes. An initial analytical framework was developed based on the identified themes and subthemes (Table 4). The commonalities and differences experienced by both health care providers and GDM diagnosed women were further analysed. At this stage, we invited participants (diagnosed women and health care providers) to provide feedback on the findings. This was to ensure that participants own meanings and perspectives were represented correctly and to avoid researcher bias creeping in. QSR International NVivo 9.0, a qualitative data storage, management and analysis software was used to identify categories and key themes and sub-themes. A thick description explains how patient-provider relationship, acceptability, participant agency and response influenced GDM management. The data is explained using both an insider view as well as an outsider view, defined as ‘emic’ and ‘etic’ perspectives in ethnographic research. As is the practice in qualitative research, pseudonyms are used throughout the manuscript for verbatim quotes to illustrate significant experiences.

**Table 4:** Identified themes and sub-themes

Major themes	Sub-themes
Heterogeneity of South Asian women	• Diversity in language, religion, practices and attitudes; but universality in ‘lack of self-care’
Health care providers: Major challenges ○ Information provision ○ Providing optimal GDM care	• Busy clinics, no time to individualise information • Difficulty managing vegetarians • Difficulty emphasising importance of exercise in pregnancy
Women: Major challenges ○ Information on lifestyle modification • Food • Exercise • Lack of culture specific advice	• Confusion – lifestyle messages unclear ▪ Relating to food types, portion size, cooking methods, timing ▪ Exercise in pregnancy • One size fits all approach – no advice pertaining to women’s cultural and regional food consumption patterns
Women’s GDM experiences: ○ GDM controlling pregnancy	• Constant state of hunger • Pregnancy experience related to checking blood sugars all the time • Increasing diagnosis of GDM in pregnancy

## Results

Typically, women consumed South Asian foods after migration and continued doing so in pregnancy, before GDM diagnosis. Barring a few, women reported consuming “our kind of food” at breakfast, lunch, and dinner, which invariably included ‘paratha or roti with vegetable or meat curries, daal, raita, curd, achar, dosai/idli, upma, rice, sambhar, curries, chutneys’ etc. Snack-time foods included deep-fried samosas, pakoras, vadais, and sweets. The second-generation participant too continued with her typical South Asian diet, but cut down on deep-fried snacks, sweets and controlled carbohydrate intake. With few exceptions, after GDM diagnosis women were reluctant to give up “our kind of food”, but nonetheless made changes in order to ensure that their baby was healthy in-utero. GDM diagnosed women primarily reduced their carbohydrate (rice and roti) intake, and completely cut out sugar and sweets from their diet. Women were unable to comprehend and accept that South Asian staple, such as rice and roti, were detrimental to their health and GDM management. They however, made the minimal required changes (reducing the quantity of food intake) to protect their baby, which led to being in a ‘constant state of hunger’.

### Heterogeneity of South Asian women

Health care providers struggled to provide information on ‘how to successfully self-manage GDM’ in a manner that women could easily understand. Providers felt the diversity of South Asian women in terms of language, culture, and religion were a major challenge in providing self-management information in group sessions.

The endocrinologists and dietitians felt that despite being diverse these women had one similarity – they did not pay “due importance to their own health needs, were too respectful, reticent, and timid to their own detriment, and did not speak up” at consultations. Providers perceived this lack of ‘self-care’ among South Asian women to be a barrier which prevented women from attaching appropriate significance to their health and condition. Moreover, providers reported that women were unable to prioritise information, and hence were unable to manage their blood glucose levels (BGL).

### Information provision by health care professionals: major challenges

Post diagnosis women attended a group health education session at respective maternity hospitals where information on GDM, health consequences, risks to mother and baby, self-management including BGL monitoring, insulin injecting technique, diet, lifestyle and exercise messages were conveyed (Table 5). At this session, women were also required to complete all necessary paperwork for the Australian Government administered National Diabetes Services Scheme (NDSS). The NDSS delivers education and information services to people with diabetes and provides a range of diabetes products at a subsidised cost. These sessions were long and lasted for two or more hours.

**Table 5:** Gestational diabetes education sessions at study hospitals for newly diagnosed women

Study hospitals	Education type	Education content
Hospital A	Individual and small group education sessions	1st session (2–3 days post-diagnosis) With a Diabetes Nurse Educator on what is gestational diabetes and how to test, record readings, monitor and manage 2^nd^ session (7–10 days post diagnosis) With a Dietitian along with a record book/diary of Blood Glucose Level (BGL) readings. Based on the record book – advice provided on importance of healthy eating, nutrition in pregnancy, and a target range of BGL maintenance; and how to maintain with diet and exercise
Hospital B	Split group sessions of 2 + hours English speaking and Non-English speaking with interpreters	Topics covered: What is gestational diabetes mellitus and self-management of condition including testing, reading, recording, and monitoring of BGLs, and how to use glucometer Importance of diet and exercise in management of the condition. Broad information on nutrition in pregnancy, healthy eating – information on carbohydrate, sugar and fat intake
Hospital C	Group session 2 + hours long Non-English-speaking women are seen individually with interpreters	1st hour on what is gestational diabetes mellitus with a Diabetes Nurse Educator; and 2nd hour on importance of healthy eating and exercise in pregnancy with a Dietitian

### Women ‘confused’ by lifestyle modification advice

Priya – a recent Indian immigrant and IT professional, stated that she was “too dazed” after her diagnosis, nevertheless, attended the information session and ‘listened’ to all they said. But when she went home, “I kind of like, couldn’t remember what I was supposed to do! All I could remember was walk after every meal and healthy eating. Only after a couple of weeks of talking to the diabetes educator and the doctor I understood how to maintain my BGL, but by then I was already on insulin”.

The diagnosed women attending education sessions were stunned at being told to reduce their food intake and exercise in pregnancy. This advice directly conflicted with their “cultural practices and beliefs about food consumption and exercise in pregnancy”. This confusion led to women being emotionally overwhelmed when asked to reduce intake of rice, roti, oils and fats. Lisa’s account is demonstrative of women’s emotions when asked to reduce food intake and to switch over to ‘other kinds’ of foods and methods of cooking. “The food we eat is different to what they eat here, and what we are told is that all this “English” food is good and “ours is not”. For us following this is hard, because people here eat steamed vegetables and meat and all those sorts of things. But we are used to eating different kinds of things. All this is too difficult you know; we are already going through the stress of having GDM, and then we have to now change our food habits as well….”

### Busy clinics and insufficient consultation time

Women expressed considerable dissatisfaction with the information messages they received from dietitians and said, ‘the session with the dietitian was not helpful’. They felt that the dietitians were unaware about “our food and culture”, as they lacked understanding of the importance of food to Asians especially in pregnancy. All they know is that “we eat rice and curries”, that is all. Women felt that if dietitians were aware and knowledgeable about the type of food South Asians ate, and particularly in pregnancy, it would be easier for women to communicate with them. On the other hand, health care providers acknowledged that insufficient time was available to individualise advice, considering the diversity that existed in women’s food preferences and exercise practices and attitudes. They perceived that because of insufficient time spent with individual women, perhaps women were unable to self-manage their condition. Also, health care providers felt that it was difficult to exactly ascertain whether women were genuinely having difficulty managing with diet and exercise, because at consultation women maintained that ‘they are doing all the right things, but still had problems maintaining optimum BGLs’.

### ‘One size fits all approach’ information provision

Women believed that if information related to diet was provided specifically on foods they should eat and foods they should avoid it would be helpful. The current advice they felt simply stated to observe a healthy diet, which included steaming vegetables, grilling or baking meat and fish, and less frying, and fewer carbohydrates. Women reported that they had no idea how to incorporate these methods of cooking into their traditional diet. “It is easy for them to say, ‘steam your vegetables and grill your meats’, but I have no idea how to prepare these” said Meena.

All the women in the study felt that information provision on lifestyle modification should be directed at the ‘patient’s culture and where they come from’ rather than a ‘one size fits all’ approach—the current mode of delivering health promotion and education messages. Some women suggested that providing simple easy to use recipes would be a good starting point, as they were always in doubt if they were doing the right thing.

“if there’s a booklet or something for people from the Indian sub-continent, like if somebody who knows exactly what we really eat and our type of food, it will be really good for us to get that when we are diagnosed and attend the education session. You know, our style of cooking is so different. So, if I can have information on how to cook, and about low Glycemic Index (GI) Indian foods, it will be really very helpful. Then I don’t have to experiment with everything I eat. I think we need something specific for our kind of diet with examples of food and what will lower our BGL”.

“Currently everything is a matter of trial and error for us. If we can get a bit more personalised care, it would be very helpful. I believe that information messages should be customised to our kind of food and to our needs. If they can provide a bit more customised diet plan for us, like if there’s a kind of printed material like the kind of food we have, like specially the roti and stuff, those which are high in carbohydrates, what should be the replacement and stuff would be great”.

The care providers at the maternity hospitals need to familiarise themselves with South Asian women’s practices in pregnancy to enable provision of culturally specific lifestyle modification advice for self-management.

### Managing vegetarians & portion size

Health care providers felt it was hard to manage vegetarian South-Asian women because their traditional staple diet composition was high in carbohydrate and fat content. The below quote is illustrative of their challenge.

“Managing vegetarians is hard, as we have to work with what women usually eat. Women from the Indian sub-continent – they normally stack up their carbohydrates on top of each other – they will have rice or roti and potatoes in the curry, or rice and daal in one meal. The oil content in their food is high as well, and deep-fried foods. It is a challenge for us to get them to reduce carbohydrates and oil from their diet. We advise them to not eat in huge quantities, restricting rice and roti intake, snacking in between meals, and re-distributing carbohydrates. But women come back saying they are hungry when they reduce their carbohydrate intake.”

Besides managing vegetarians, another significant challenge was communication about portion size. Weight management was a major problem according to the dietitians.

“By the time women attend the education session after GDM diagnosis (post 26-28 weeks of pregnancy), they are significantly heavier by around 20-25 kilograms owing to their diet and sedentary lifestyle”. At the education session “when I show them the measuring cup and the portion size/serve they are supposed to have, they are amazed and say that ‘it is too little’.”

Women and their family members believed that food restriction/control would affect their baby’s growth and development.

“Restricting and controlling my diet was very difficult. I was not having that much food, and you know, I was deprived of having good food, and half the time, I was hungry. I think I didn’t gain any weight nor did the baby. First, he was of average weight, and then he didn’t gain weight. So, what I think - because I restricted my diet you know - not having carbohydrates and stuff and you know, this dietary stuff. Then the sugars were okay, not too good still. Then when I had the scan and they figured out that you know, the baby is not gaining that much weight, I was very worried. So, after that I didn’t restrict myself, the sugars went up, and I was on insulin, now I am on insulin and I am eating so you know, things are fine”.

This quotation is illustrative of the importance of cultural beliefs and practices surrounding pregnancy health and wellbeing for the participants.

### Being physically active: a cultural clash of ideas about physical activity in pregnancy

Another challenge for both providers and women was related to being physically active in pregnancy. Health care providers were unable to emphasise the importance of being physically active in pregnancy to women and their family. They commented that it ‘becomes challenging to get women moving, as most South Asian women in Australia avoid doing ‘too much’ physical activity in pregnancy’. At the maternity hospitals standard physical activity advice is provided, which is ‘brisk walk for at least 30 min a day’.

### Losing control of the pregnancy

Women talked at length about ‘losing control over their pregnancy’ and how maintaining targeted BGL dominated their pregnancy experience.

“It’s like your mind is always occupied with diet and walking and measuring the glucose level! I can’t go out; I feel like I can’t go out anywhere or just enjoy anything anymore. I am inside the house, all the time, I use the treadmill and all my concentration is on what I eat, walk, how much I eat, walk, and eat, walk, eat, walk, that’s it, I don’t do anything else. My experience with this pregnancy is all about eating, walking, and checking my glucose.”

Most of the women in the study were on insulin as they were unable to self-manage their condition. A few women who had completely stopped their traditional diet and method of cooking were very dissatisfied with their pregnancy experience and viewed having GDM as a punishment/curse. They frequently commented that they were concerned about the increasing numbers of South Asian women being diagnosed with GDM in Australia and questioned if pregnancy was over medicalized in Australia.

“I noticed one thing, like I don’t think it's a question, but most of my friends, like they're all Indian, and they came here, and they were having their first babies, and all have been diagnosed with gestational diabetes! So, I mean that’s, I don't know, are people aware or doctors aware they're overprotective? I mean, I told my mum that I am checking my sugar four times a day and I’ve been put on insulin. She was like quite surprised and quite concerned, because in India I mean you don’t really see, I mean women being pregnant and put on insulin. It was like very stressful for me after my diagnosis like with no family support and working full-time”.

Women felt that GDM was controlling their pregnancy experience. Since diagnosis it was all about checking blood glucose after each meal, being afraid of food, being in a ‘constant state of hunger’, feeling depressed and anxious, and yet unable to control the sugar levels despite doing what was suggested at consultations. The second-generation participant too felt that GDM was controlling her pregnancy; but with family support (both maternal family and in-laws) she was able to modify her lifestyle to manage her BGL.

## Discussion

The heterogeneity of South Asian women proved to be a major challenge for health care providers in successfully delivering health messages for self-management. Although South Asia has varied languages, ethnic groups, religions, and culture, they however are universal in terms of food habits, food preparation and methods of cooking. They are also similar in their beliefs and practices around pregnancy and pregnancy health, childbirth, labour and the postpartum period [8]. The second-generation woman too followed traditional practices in pregnancy.

Proper management of GDM during pregnancy leads to better health outcomes for both mother and child [16, 17]. With increasing migration from South Asia into Australia, and the high incidence of GDM among South Asian women, this study raises important issues concerning current care of these women. The main area of concern is the mismatch between health care provider’s messages about diet and physical activity and the capacity of South Asian women to understand and incorporate these into their daily lives. The messages do get ‘lost in translation’.

Maintaining normal blood glucose levels is important during pregnancy and labour and therefore self-monitoring of blood glucose levels by women is a key daily practice in good quality management. However, women from South Asia [8] found it challenging to self-manage their GDM. Women reported that the information provided on lifestyle modification (diet and exercise) for self-management was culturally and socially inappropriate and irrelevant to their needs. Messages provided were often confusing for women leading them to explore alternative sources to find relevant information [18] to maintain their cultural practices in pregnancy, particularly diet-related.

In pregnancy, in South Asia one is supposed to eat for two [19] and restrict strenuous physical activity and gain weight. Not only did women have to come to terms with their diagnosis, but also had to make drastic changes to their dietary habits and lifestyle. In most of South Asia the socioeconomically disadvantaged women have adverse pregnancy outcomes, hence the affluent expectant mothers are encouraged to eat for two and rest for favourbale pregnancy outcomes [8]. Post diagnosis in Australia, these women felt trapped between two diverse cultural notions of pregnancy health and weight. Women were not only coping with the diagnosis but were also asked to make massive dietary and lifestyle changes, contrary to their pregnancy beliefs and practices [8]. The second-generation participant continued with her traditional South Asian diet; but made certain lifestyle modifications to manage her condition without insulin.

Health care providers involved in the care and treatment of South Asian women were aware that the education messages were not understood nor well-received and were viewed as threatening and punishing. GDM self-management messages should be provided in such a way so that women are able to maintain and sustain their traditional diet in pregnancy. Providing culturally specific lifestyle modification advice for self-management would lessen the burden on health staff and minimize repeated hospital visits and insulin dependence.

A previous [8] study informed the development of sample menus for GDM diagnosed South Asian women based on the types of foods eaten before, during, and after pregnancy to promote healthy living [20]. Some health care providers felt that ‘it was a big ask’ for women to immediately change their lifestyle ‘so completely’ when diagnosed at 28–30 weeks gestation. They felt that women needed more time to adjust their behaviour—“if women had more time to absorb messages about GDM, it would work better”.

Type 2 diabetes studies have shown that for patient-mediated interventions to be beneficial it is important to engage patients in effectively self-managing their condition and promoting self-management strategies [21, 22]. A study in Australia found that South Asian women were prepared to modify lifestyle for optimal fetal health and development [8]. This was found in other studies [23] which suggested that women were motivated to maximize their children’s health and adopt healthy behavioural practices in pregnancy [24].

Generally, self-care is not a priority for South Asian women, as they themselves place a low priority on their own needs and focus on the needs of their family, often neglecting or overlooking their own well-being. Certain customs and traditions perpetuate this view, affording women a low status in society [25] and this consequently influences women’s comprehension of the seriousness of their condition. This observation was pertinent to the women in this study. As self-management of BGL forms the crux of GDM management in pregnancy, it is necessary to find novel ways of engaging South Asian women diagnosed with GDM for effective self-management.

Large group information sessions (all diagnosed women irrespective of English proficiency) as existed in the study hospitals are less than ideal, and small group sessions (groups split according to English proficiency) have in fact been found to be both effective and cost-effective in the management of GDM [26]. Both South Asian women and their health care providers perceive the current information/education provision to be unsatisfactory and limiting. Therefore, there is an urgent need to modify GDM information provision to meet the needs of South Asian women.

Daily life for most South Asian women does not include a focus on exercising for the sake of exercise and during pregnancy physical activity may be restricted because of cultural beliefs around pregnancy [8]. Getting women moving and being active after GDM diagnosis is critical in maintaining BGL, so concerted efforts are needed to assist South Asian women to incorporate more physical activity into their daily routines. This requires working with each woman individually to identify areas where she can make acceptable changes. Diabetes studies have demonstrated that diabetes remained lowest in the lifestyle group [27], and that the risk of type 2 diabetes can be prevented by changes in the lifestyles of high-risk subjects [28]. Studies have also confirmed that mild to moderate lifestyle intervention had a major impact in preventing diabetes among impaired glucose tolerant subjects [29–32]. Evidence suggests that clearly illustrated diet plans with portion size of meals, including low GI foods, weekly meal plans, and physical activity suggestions such as daily walks/exercise, are essential and useful tools that aid in lifestyle modifications [20, 33, 34]. Therefore, the importance of lifestyle intervention messages should promote ‘incidental physical activity’ that can be integrated into women’s daily activities.

Studies have demonstrated the significant increase in total health care costs for treating women on insulin compared to women managing their condition by lifestyle interventions [35, 36]. But a recent study found a twice-weekly exercise program for pregnant women at risk of GDM was not cost-effective compared to standard care [37]. Nonetheless, health care costs are higher for overweight or obese pregnant women and for women at risk of GDM than for women with normal weight [38, 39].

Health promotion studies have shown that beliefs about health are likely to have an impact on health behaviour [40] and how one defines health varies by cultural background, thus influencing health practices [41]. As identified in this study, lifestyle management is particularly difficult for South Asian women with GDM as most were recent immigrants and did not have support of family. The second-generation participant with the support and help from her family was able to self-manage her condition. Therefore, concerted efforts are needed that are responsive to women’s actual circumstances. Knowledge about the different regional food preferences (vegetarians, vegans, pescatarians), patterns and habits (eating times of main meal, special foods for different occasions, food preparation as per religious belief, use of ghee, oil) and pregnancy rituals of the sub-continent; and an understanding of how to manage GDM with a vegetarian diet is fundamental, but currently lacking.

Health care providers agreed that they were unable to deliver health education messages in a culturally specific manner. They were aware that to facilitate behaviour change, health messages should be tailored to the health beliefs, and be precise and to the point [40]. When health messages and/or therapeutic instructions are adapted to the social and cultural background as well as to individual needs, it has been shown that women with GDM from a range of ethnicities can achieve comparable outcomes [42].

### Strengths and limitations

This study provides an in-depth analysis of women’s narratives of their experiences, and their health care providers perspectives, presenting an insider’s view of both patient and health care provider’s social worlds including challenges and practices. It presents the experience of a second-generation South Asian woman – to my knowledge no previous studies have reported. A limitation of this study is that it may not be generalisable to other immigrant groups in Australia or elsewhere but is representative of South Asian immigrant women’s experiences [43] and women of similar ethnic backgrounds. Health care providers perspectives maybe generalisable in the same context of the study but may not be applicable to all health care providers in Australia or elsewhere. This study generates interesting insights and provides a holistic way of exploring relationships [44–46] and forms the basis for other studies to explore how universal is the phenomenon in other settings.

### Implications for practice

This study has a number of important implications for practice, as rates of GDM continue to rise globally and in Australia [47]. Women from the Indian sub-continent constitutes a growing group of parturient women in Australia and are at particularly high risk of developing GDM and subsequently Type 2 diabetes [29, 31, 32]. Information on GDM is not available in South Asian languages suggesting that there is an urgent need to rectify this to reduce the burden of GDM among women from this group. An additional difficulty is the limited information available on culturally appropriate modifications of their usual diet in South Asian languages. These challenges could be addressed by providing relevant information to health care professionals on the prevalent diversity of diets in South Asia, healthy behavioural practices in pregnancy for optimal fetal health and development—as South Asian women are motivated to maximize their children’s health [24]. Targeted educational resources in simple and clear language and format that are culturally appropriate and include acceptable exercise recommendations and dietary modifications to usual South Asian foods eaten should be given at antenatal visits. Repeated information and advice targeting not only women, but their family is likely to be the most successful in preventing Type 2 diabetes in later life. The findings of this study are likely to be still relevant as the model of GDM management and care in pregnancy focuses on optimal management with diet and exercise to control blood sugars.

## Conclusions

The current clinical practice does not meet expectations in an area where self-management behaviour forms the crux of GDM management at the study hospitals. Health care providers need to be aware of diverse patient cultures, rather than applying the current generic approach which fails to engage and meet the needs of immigrant and ethnic women at the study hospitals. Future enabling strategies should include developing, evaluating, and disseminating appropriate diet plans, models and resources for use with South Asian women diagnosed with GDM.

## Supplementary Information

**Additional file 1.**

**Additional file 2.**

## Data Availability

This manuscript is based on qualitative data. Data sharing is not applicable as no datasets were generated.

## Abbreviations

GDM: Gestational diabetes mellitus
BGL: Blood glucose level
OGTT: Oral glucose tolerance test
DNE: Diabetes nurse educator
