# Supplementary material for: Gestational diabetes mellitus: a qualitative study of lived experiences of South Asian immigrant women and perspectives of their health care providers in Melbourne, Australia
Source: BMC Pregnancy Childbirth. 2021 Jul 9;21:500. doi: 10.1186/s12884-021-03981-5 (PMC8272384; doi:10.1186/s12884-021-03981-5)
Supplement: Supplementary file 1 — Additional file 1. [file 12884_2021_3981_MOESM1_ESM.doc]

### Interview guide for Women

Interviews are useful for getting the story behind a participant's experiences. Before starting the interview, the interviewer will explain the purpose of the interview, address terms of confidentiality, and explain who will have access to their answers and how their answers will be analysed. The format and nature of the interview will be explained and how long the interview might take. The interviewer will tell participants how to get in touch with the research team later if they want to. The participants will be asked if they have any questions before the interview starts, and permission to record the interview will be sought. Interviews will begin with some facts as the respondents can more easily engage in the interview before warming up to more personal matters. The wording of the interview will be open-ended and women will be invited and encouraged to tell their stories.

### (24-28 weeks gestation during pregnancy)

- General socio-demographic information including age, education, occupation, migration status, length of stay in Australia, current housing status, income, number of children, previous pregnancy, and so on.
- Please tell me in detail about your feelings when you were told that ‘you have developed gestational diabetes’, and your perception and understanding of gestational diabetes. (probing questions: family history of diabetes, previous pregnancy, what steps were taken previously to manage condition).
- What management strategies were communicated to you by the health care staff at the hospital? (probe: food, exercise, lifestyle changes, any difficulty in understanding what was communicated; asked any questions from health staff when in doubt).
- Are you able to successfully manage your condition? What kind of support is available to you at home? (probe: what do you do in terms of food, eat the same food that is prepared for the family, have you cut down on the recommended foodstuff, if not , please explain why; do you get time to exercise to control your condition, what are the barriers and facilitator, what type and kind of support is available at home from partner/husband, any family, or friends).
- Is this impacting on your work in and out of home? If so, how are you able to manage your health, and work? What steps are you taking to ensure a good pregnancy outcome?
- Are you taking any medication to manage your condition? What are you doing to manage your gestational diabetes if you are not on any medication? Do you get support from you partner/husband, family and friends in managing this?
- What kind of support would you require in managing GDM in pregnancy? How is it impacting on your overall wellbeing? Are you able to talk about it with your health care providers? If no, why not?
- Would you like to say anything else at all?
