# Supplementary material for: Gestational diabetes mellitus: a qualitative study of lived experiences of South Asian immigrant women and perspectives of their health care providers in Melbourne, Australia
Source: BMC Pregnancy Childbirth. 2021 Jul 9;21:500. doi: 10.1186/s12884-021-03981-5 (PMC8272384; doi:10.1186/s12884-021-03981-5)
Supplement: Supplementary file 2 — Additional file 2. [file 12884_2021_3981_MOESM2_ESM.doc]

### Interview guide for Health Care Professionals

Before starting the interview, the interviewer will explain the purpose of the interview, address terms of confidentiality, and explain who will have access to their answers and how their answers will be analysed. The format and nature of the interview will be explained and how long the interview might take. The interviewer will tell participants how to get in touch with the research team later if they want to. The participants will be asked if they have any questions before the interview starts, and permission to record the interview will be sought. Interviews will begin with some facts as the respondents can more easily engage in the interview before warming up to more personal matters. The wording of the interview will be open-ended.

**Prompts:**

- General socio-demographic information including age group, education, specialisation, occupation, years in occupation/profession, how long working with South Asian women, and so on
- Would you describe the role you have in the care of women with GDM.
- How do you find caring for women of South Asian backgrounds?
- Could you please tell me in detail if you have faced any difficulties/problems communicating with a South Asian woman diagnosed with GDM?
- Do you know anything about South Asian women’s beliefs about food preference and exercise and workload during pregnancy or other cultural practices related to pregnancy?
- How does this affect your advice on management of GDM if at all?
- Do you take any measures if women fail to take up the advice provided? How do you monitor women from the time they are diagnosed until they go into labour?
- How do you emphasise and articulate the importance of GDM and its management to women and their family/partner?
- Do you talk to women about progression to type 2 diabetes in the long-term? When do you talk to them about this? What kind of advice and health message do you convey at this stage?
- Do you have any GDM information in South Asian languages?
- Have you found any particular strategies useful in caring for South Asian women?
